# Neuron-glia cross talk revealed in reverberating networks by simultaneous extracellular recording of spikes and astrocytes' glutamate transporter and K^+^ currents

**DOI:** 10.1152/jn.00509.2016

**Published:** 2016-09-28

**Authors:** Enzo Wanke, Francesca Gullo, Elena Dossi, Gaetano Valenza, Andrea Becchetti

**Affiliations:** ^1^Department of Biotechnologies and Biosciences and Milan Center For Neuroscience (NeuroMI), University of Milano-Bicocca, Milan, Italy; and; ^2^Research Centre “E. Piaggio” and Department of Information Engineering, School of Engineering, University of Pisa, Pisa, Italy

**Keywords:** AraC, MEA, neocortex, K^+^ buffering, TBOA

## Abstract

Astrocytes uptake synaptically released glutamate with electrogenic transporters (GluT) and buffer the spike-dependent extracellular K^+^ excess with background K^+^ channels. We studied neuronal spikes and the slower astrocytic signals on reverberating neocortical cultures and organotypic slices from mouse brains. Spike trains and glial responses were simultaneously captured from individual sites of multielectrode arrays (MEA) by splitting the recorded traces into appropriate filters and reconstructing the original signal by deconvolution. GluT currents were identified by using dl-threo-β-benzyloxyaspartate (TBOA). K^+^ currents were blocked by 30 μM Ba^2+^, suggesting a major contribution of inwardly rectifying K^+^ currents. Both types of current were tightly correlated with the spike rate, and their astrocytic origin was tested in primary cultures by blocking glial proliferation with cytosine β-d-arabinofuranoside (AraC). The spike-related, time-locked inward and outward K^+^ currents in different regions of the astrocyte syncytium were consistent with the assumptions of the spatial K^+^ buffering model. In organotypic slices from ventral tegmental area and prefrontal cortex, the GluT current amplitudes exceeded those observed in primary cultures by several orders of magnitude, which allowed to directly measure transporter currents with a single electrode. Simultaneously measuring cell signals displaying widely different amplitudes and kinetics will help clarify the neuron-glia interplay and make it possible to follow the cross talk between different cell types in excitable as well as nonexcitable tissue.

## Abstract (precis)

*In neocortex networks, we simultaneously captured spikes and the slower astrocytes' K*^+^ *and glutamate transporter (GluT) currents with the use of individual MEA electrodes. Inward and outward K*^+^ *currents in different regions of the glial syncytium suggested that spatial buffering was operant. Moreover, in organotypic slices from ventral tegmental area and prefrontal cortex, the large GluT current amplitudes allowed to measure transporter currents with a single electrode. Our method allows direct study of the dynamic interplay of different cell types in excitable and nonexcitable tissue*.

## NEW & NOTEWORTHY

*In neocortex networks, we simultaneously captured spikes and the slower astrocytes' K*^+^ *and glutamate transporter (GluT) currents with the use of individual MEA electrodes. Inward and outward K*^+^ *currents in different regions of the glial syncytium suggested that spatial buffering was operant. Moreover, in organotypic slices from ventral tegmental area and prefrontal cortex, the large GluT current amplitudes allowed to measure transporter currents with a single electrode. Our method allows direct study of the dynamic interplay of different cell types in excitable and nonexcitable tissue*.

neuron-astrocyte signaling is a classic example of cell-cell communication in a complex tissue, with manifold pathological implications (Robel and Sontheimer 2016; Xiong et al. 2016). Astrocytes support neuronal metabolism (Choi et al. 2012; Hassel et al. 1995; Paulsen et al. 1987; Rosenberg et al. 1992; Rouach et al. 2008) and prevent extracellular accumulation of neurotransmitters, especially glutamate. In neocortex and hippocampus, astrocytes express two types of electrogenic glutamate transporters: GLT-1 and GLAST (Danbolt 2001; Huang and Bergles 2004; Lehre et al. 1995). Although in mature tissue GLT-1 is thought to prevail (Haugeto et al. 1996), both transporters are significantly expressed during neocortex development (Hanson et al. 2015). Moreover, classic work in nonmammalian nervous tissue showed that astrocytes buffer the spike-dependent extracellular K^+^ ([K^+^]_o_) excess that cannot be quickly handled by the Na^+^ pump (Frankenhaeuser and Hodgkin 1956; Kofuji and Newman 2004; Newman et al. 1984; Orkand et al. 1966). In agreement with earlier work, simultaneous in vivo recording from pyramidal neurons and astrocytes in cat cerebral cortex showed that each spike train triggers a delayed, time-locked astrocyte depolarization (Amzica and Neckelmann 1999). In particular, in brain regions ranging from hippocampus (Bergles and Jahr 1997; Diamond et al. 1998; Ge and Duan 2007; Henneberger and Rusakov 2012; Lüscher et al. 1998) to olfactory bulb (De Saint Jan and Westbrook 2005), afferent stimulation evokes astrocyte inward currents comprising a fast and a long-lasting component. These are mediated, respectively, by GluTs (mainly GLT-1; Danbolt 2001; Huang and Bergles 2004) and K^+^ channels. The full complement of K^+^ channels expressed by astrocytes is unclear (Olsen et al. 2015). The main contribution to K^+^ buffering is thought to be provided by weakly rectifying Kir4.1 channels (Djukic et al. 2007; Kucheryavykh et al. 2007; Olsen et al. 2006; Seifert et al. 2009). The role of other K^+^ channels such as the two-pore domain channels is debated (Du et al. 2016; Hwang et al. 2014; Olsen et al. 2015; Zhou et al. 2009).

Although patch-clamp methods provide the sensitivity required to record transporter currents, they are generally unsuitable to study the global activity of a wide excitable network, and thus to correlate neuronal spiking with the response of an ensemble of glial cells. To solve these issues, we adapted multielectrode array (MEA) recording to simultaneously study the astrocyte and neuronal currents throughout a neocortical network. Field potential amplitudes depend on the current source density and steeply decrease with distance (Buzsáki et al. 2012; Holt and Koch 1999; Pettersen and Einevoll 2008). Therefore, although each MEA electrode is surrounded by ∼50 neurons (within a 100-μm-diameter circle), only the activity of the few neurons closest to the electrode can be reliably acquired (Becchetti et al. 2012; Gullo et al. 2009, 2010, 2014a; Puia et al. 2012). In contrast, because a few astrocytes can cover an area much larger than that occupied by 50 neurons (Bushong et al. 2002), a sparse electrode resolution (∼200 μm) should be sufficient to record field potentials from the astrocytes surrounding a firing neuron (Dossi et al. 2013). Hence, shortly after each network burst, one expects to record the delayed negative-going signals caused by astrocyte K^+^ currents, with a rise time similar to that displayed by astrocyte depolarization in vivo (Amzica and Neckelmann 1999).

By exploiting the different kinetics of action potentials and astrocyte responses, we developed an acquisition/analysis procedure to distinguish these events in neocortex networks expressing a physiological ratio of neurons and glial cells (Dossi et al. 2013; Gullo et al. 2010, 2014a). During network bursting, we could record from each electrode the neuronal spikes, the relatively fast inward GluT currents, and the slower K^+^ currents. These methods should greatly facilitate the investigation of K^+^ and neurotransmitter reabsorption in wide neuronal networks. Moreover, they may be extended to the study of cell-cell and cell-microenvironment interaction in excitable and nonexcitable tissues, in normal as well as pathological conditions.

## MATERIALS AND METHODS

### 

#### Chemicals and drugs.

Unless otherwise indicated, chemicals and drugs were purchased from Sigma-Aldrich (Milan, Italy). Stock solutions of tetrodotoxin (TTX; Tocris Bioscience, Bristol, UK), 2-(3-carboxypropyl)-6-(4-methoxyphenyl)-2,3-dihydropyrazidin-3-iminium bromide (gabazine), dl-threo-β-benzyloxyaspartate (TBOA; Tocris Bioscience), and cytosine β-d-arabinofuranoside (AraC) were prepared in distilled water and stored at −20°C. On the day of the experiment, stock solutions were diluted at their final concentration with our culture medium.

#### Primary cultures.

Mice were manipulated according to the Principles of Laboratory Animal Care (directive 86/609/EEC). Our procedures were prospectively approved by the Italian Ministry of Health. Cerebral cortices (excluding the hippocampus) were extracted from decapitated FVB postnatal *day 1–3* (P1–P3) mice (Charles River, Calco, Italy). Primary cultures were prepared as previously described (Gullo et al. 2009) and laid onto MEA dishes coated with polyethyleneimine (0.1% wt/vol) and laminin (20 μg/ml). Cultures were covered with gas-permeable covers (MEA-MEM; Ala Scientific Instruments, Farmingdale, NY) throughout the culture period and kept at 37°C in 5% CO_2_. One-half of the medium was replaced every 3 days. AraC was applied at 5 μM when the plating medium was replaced. After 2 days in vitro (DIV), the concentration was increased to 10 μM; after 6 DIV, it was increased to 20 μM.

#### Organotypic cultures.

Organotypic slice cocultures were prepared from FVB P1–P2 mice (Charles River). Coronal sections (200 μm thick) were cut from ventral tegmental area (VTA) and prefrontal cortex (PFC), prepared as previously described (Dossi et al. 2013), and maintained at 37°C in 5% CO_2_. After 2 DIV, the incubation medium was replaced with a serum-free medium consisting of Neurobasal medium supplemented with B27 (Invitrogen), glutamine (1 mM), and penicillin-streptomycin (150 μg/ml).

#### MEA recording and waveform acquisition.

**Fig. 1.**
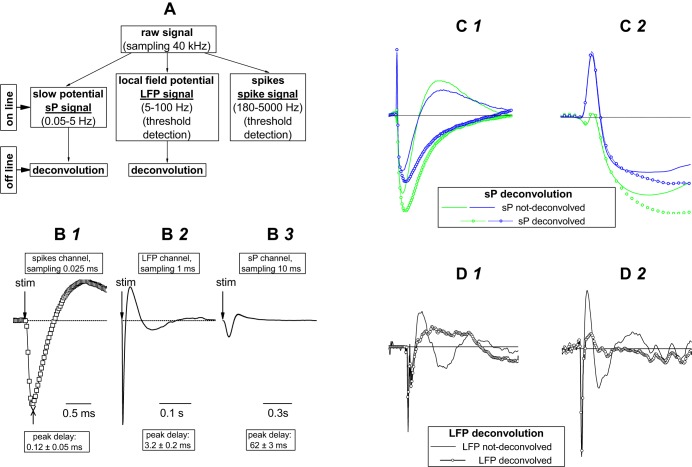
Acquisition and deconvolution of slow potential (sP) and local field potential (LFP) waveforms. *A*: the raw signals were split online into 3 different filters whose output essentially constituted sPs, LFPs, and spikes, as indicated. Waveform distortion by filtering was corrected by a deconvolution procedure. *B1–B3*: the impulse responses of the filters (*B1*, open squares; *B2* and *B3*, lines) were obtained by injecting a 0.1-ms pulse into the microelectrode array (MEA) chamber and then recording the corresponding response from each channel. *C1*: representative sP signals (blue and green lines) are superimposed to the corresponding deconvolved waveforms (circles). *C2*: the early part of *C1* is expanded to show that the fast portion of the original sP signal was not modified by deconvolution. *D1* and *D2*: representative LFP signals (lines) and their deconvolved waveforms (circles). Deconvolution largely removed the spurious oscillation observed in the output of sP and LFP channels.

Data acquisition and analysis were carried out as previously described (Dossi et al. 2013; Gullo et al. 2009); we specify in this report the changes we introduced thereafter. Analog signals were recorded at 36°C in CO_2_-controlled incubators using either 60 (MEA-1060BC)- or 252-electrode MEA amplifiers (Multichannel Systems MCS, Reutlingen, Germany). Electrodes were connected to the Multichannel acquisition system hardware and, when necessary, also to a 60-electrode Plexon MEA workstation (bandwidth 100-8,000 Hz; Plexon, Dallas, TX). As shown in Fig. 1*A*, raw data were sampled at 40 kHz by using MC_Rack software (version 4.0.4; MCS). The ensuing signal was split online into three distinct channels: one for spikes, one for local field potentials (LFPs), and one for slow potentials (sPs). Spikes were acquired by applying a 4-pole high-pass Bessel filter at 200 Hz, with the following threshold detection parameters: pretrigger, 1 ms; posttrigger, 1 ms; dead time, 1 ms. The LFP data were acquired from 5 to 100 Hz (4-pole Bessel filter), with pretrigger set at 100 ms, posttrigger at 500 ms, and dead time at 500 ms (sampling rate was 1 kHz). This setting was chosen to capture the GluT currents, based on previous studies (Bergles and Jahr 1997; De Saint Jan and Westbrook 2005; Diamond et al. 1998; Ge and Duan 2007; Henneberger and Rusakov 2012; Lüscher et al. 1998). The sP channel data were filtered at 5 Hz (4-pole Bessel filter) and continuously acquired with a sampling rate of 100 Hz to capture the K^+^ astrocyte currents, according to the features of glial depolarization observed in amphibian glia (Orkand et al. 1966) and cortical astrocytes (Amzica and Neckelmann 1999). The MCS amplifier was factory-modified to extend the low-frequency band up to 0.05 Hz with the addition of a double RC filter.

#### Neuronal and burst cluster classification.

**Fig. 2.**
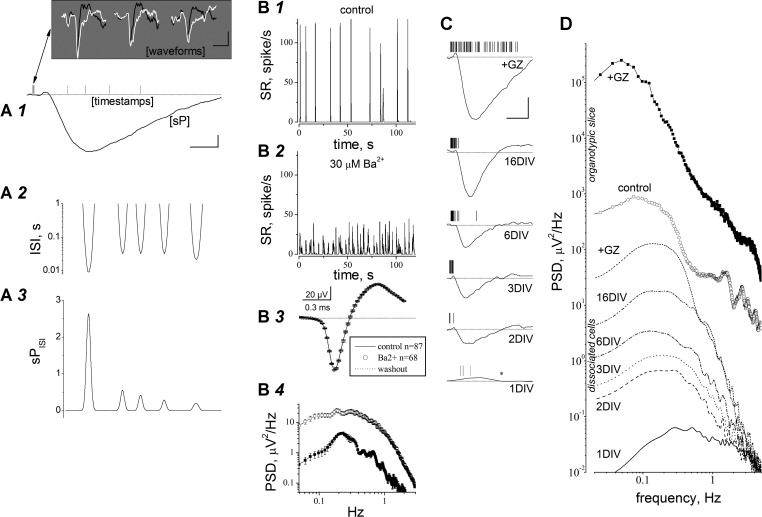
sP signals are Ba^2+^-sensitive K^+^ currents in primary cultures and organotypic slices. *A1*: representative sP signal (continuous line) and aligned spike timestamp*s*. Scale bars, 5 μV and 0.2 s. The first 3 spikes are expanded in the *inset* (scale bars, 30 μV and 1 ms). The black and white traces were obtained, respectively, after high-pass filtering with bandwidths of 120-8,000 and 5–8,000 Hz. *A2*: interspike interval (ISI) plot relative to the sP waveform in *A1* but right-shifted by 0.4 s. *A3*: plot of sP divided by ISI (sP_ISI_), aligned to the sP in *A1*. *B1* and *B2*: time course of spike rate (SR) in control (*B1*) and in the presence of 30 μM Ba^2+^ (*B2*) during 120 s of activity (bin, 5 ms; running average with 15-point smoothing). Ba^2+^ decreased the peaks of activity from 100–125 spikes/s to less than 50 spike/s. *B3*: superimposed spikes in control (line), in the presence of 30 μM Ba^2+^ (circles), and after washout (dotted line). Data are averages of the indicated number of traces. *B4*: power spectral density (PSD) computed in control (open circles) and in the presence of 30 μM Ba^2+^ (for 10 min; filled circles). Data are averages of the responses of 6 electrodes in a representative experiment (1 of 4). *C*: sP traces recorded from the same electrode at the indicated number of days in vitro (DIV) with the corresponding spike timestamps. Notice the sP increase with age and the effect of gabazine (GZ; at 16 DIV, 3 μM). Scale bars, 10 μV and 0.5 s. *D*: PSD analysis of sP signals at different times in vitro for primary cultures (lines) and organotypic slices (lines + symbols). GZ was added at 16 DIV. The organotypic slice data are averages from 6 electrodes sampling a prefrontal cortex (PFC) area for 300 s, at 15 DIV, from an MEA256 dish (1 of 4). Dissociated cells were analyzed in the same electrode of a typical MEA dish (1 of 8), at the indicated DIV (data are averages of 5 consecutive bursts).

For each unit, we computed burst duration (BD), spike number (SN), intraburst spike rate (IBSR), Fano factor (FF; time window of 6 s), interspike interval (ISI), squared coefficient of variation (CV^2^; computed from ISI histograms), and intraburst intervals (IBIs). Activity bursts were detected and classified as reported previously (Becchetti et al. 2012; Gullo et al. 2009, 2010, 2012). Briefly, the bursts that presented more than two spikes were identified with NeuroExplorer software. When two consecutive spikes were observed, we assigned a BD equal to their ISI, and a SN of 2. For isolated spikes, we assigned a BD of 3 ms (i.e., larger than the refractory ISI used during acquisition) and a SN of 1. The rationale for this procedure is discussed in Becchetti et al. (2012). We generally disregarded the units that fired continuously (1–2 in each network). The effectiveness of such a procedure was previously confirmed by Gullo et al. (2010), in which SN, BD and burst number data were consistent with those displayed in the present study. For each neuron, the burst data were averaged over the time segments of interest. Neurons were classified according to an unsupervised learning approach consisting of data-reducing principal component analysis, followed by a *K*-means clustering procedure. Clustering was improved by using an outlier removal procedure that discarded the units whose Mahalanobis distance from the centroid of the cluster was greater than 1.4 (Gullo et al. 2010). The program generates files associated to the two neuron clusters, giving *1*) the average spike number time histograms (SNTH), which characterize the distribution of the spikes engaged in each burst (see Fig. 7*B*), and *2*) the probability density function of finding the 1st, 2nd, 3rd, *i*-th spikes (firing spike histogram; FSH), which characterizes the firing mode of neuronal clusters. Our software and explanation text files are freely available (see endnote at the end of this article); for details, see Becchetti et al. (2012).

#### Offline deconvolution of sP and LFP signals.

To correct the waveform distortions introduced by filtering, a deconvolution process was applied. Given the acquired time dependent signals *Y*_sP_(*t*) and *Y*_LFP_(*t*) and the actual impulse responses *H*_sP_(*t*) and *H*_LFP_(*t*) of the filters used to acquire, respectively, sP and LFP signals (*Fig*. 1, *B2* and *B3*), the estimated inputs *X*_sP_(*t*) and *X*_LFP_(*t*) are respectively defined as

(1) $${\text{X}}_{\text{sP}}(\text{t})=\text{iFFT}\{{\text{X}}_{\text{sP}}(\text{f})\}\;=\;\text{iFFT}\{{\text{Y}}_{\text{sP}}(\text{f})/{\text{H}}_{\text{sP}}(\text{f})\}$$

and

(2) $${\text{X}}_{\text{LFP}}(\text{t})\;=\;\text{iFFT}\{{\text{X}}_{\text{LFP}}(\text{f})\}\;=\;\text{iFFT}\{{\text{Y}}_{\text{LFP}}(\text{f})/{\text{H}}_{\text{LFP}}(\text{f})\},$$

where iFFT is the inverse fast Fourier transform and *X*_sP_(*f*), *Y*_sP_(*f*), *H*_sP_(*f*), *X*_LFP_(*f*), *Y*_LFP_(*f*), and *H*_LFP_(*f*) refer to Fourier transform representations in the frequency domain. The impulse-response functions of the filters were obtained by injecting a 0.1-ms pulse into the MEA chamber and then recording the corresponding response from each channel (Fig. 1, *B1–B3*). The peaks of the spike, LFP, and sP signals were delayed with respect to the pulse by 0.125 ± 0.05, 2.1 ± 0.18, and 62.4 ± 2 ms, respectively (*n* = 10). These delays are consistent with the acquisition sampling rate and with the electrophysiological properties of spikes, GluT currents, and K^+^ currents, respectively. Our procedure warrants that the electrophysiological signals from both neurons and astrocytes follow the same pathway. The major distortion introduced by filtering was a decaying oscillatory behavior at ∼0.05 and 5 Hz in the sP and LFP traces, respectively. Some of the LFP components were described in Dossi et al. (2013). Moreover, although the sP signal was always delayed relative to the LFP signal, its amplitude was sometimes larger than twice the amplitude of the LFP signal, which was poorly filtered during acquisition in the 5-Hz region. In this case, we preliminarily applied fast Fourier transform filtering (high pass; cutoff at 4 Hz) to remove the putative components below 4 Hz that were not blocked by the 5-Hz high-pass Bessel filter (recall that during acquisition all of the components were mixed in the raw data channel).

#### Data analysis and statistics.

Data files were analyzed offline with NeuroExplorer. The power spectral density (PSD) data were analyzed with 4,096 frequency values, 50% Hamming window overlap, using the standard log of PSD (dB) normalization (MATLAB) that was converted to microvolts squared per Hertz (μV^2^/Hz) in the figures. All data are means ± SE, with n indicating the number of experiments (i.e., the number of MEA dishes, unless otherwise indicated). Statistical significance was assessed by carrying out the tests indicated in the text or figure legends with XLSTAT-Pro software (XLSTAT 2013.1.01; Addinsoft, New York, NY). OriginPro 7.0 software (OriginLab, Northampton, MA) was used for analysis and for preparing the figures. OriginPro 9.1 was sometimes used for deconvolution analysis.

## RESULTS

Most experiments were carried out in both primary cultures and organotypic cocultured VTA-PFC slices. The former permitted us to study the effect of blocking cell proliferation and to better distinguish the specific signals of individual cells because of the lower cell density. The latter better preserved the local connectivity and the narrow interstitial spaces typical of the central nervous system (CNS).

### 

#### sP signals reflected spike-related K^+^ currents in primary cultures.

Each spike train was usually followed by a slow negative sP trace (Fig. 2*A1*). Such sP signals *1*) were time-locked to network bursts, *2*) had peak amplitudes often larger than 1–2 mV, in mature networks, with an ∼250-ms delay relative to the burst start, *3*) were abolished by TTX (0.2 μM), and *4*) had larger amplitudes when network activity was increased by blocking the GABA_A_ receptors with gabazine (GZ; 3 μM). The sP time course resembled the reported kinetics of [K^+^]_o_ changes (Amzica and Neckelmann 1999; Frankenhaeuser and Hodgkin 1956; Orkand et al. 1966) and were consistent with Kir current kinetics (Butt and Kalsi 2006; De Saint Jan and Westbrook 2005; Maldonado et al. 2013; Sibille et al. 2014). To test the extent to which sPs depended on Kir channels, we applied 30 μM Ba^2+^, which potently blocks Kir (Butt and Kalsi 2006) but is poorly effective on two-pore K^+^ channels (Zhou et al. 2009). Ba^2+^ strongly reduced sPs without altering the spike shape, indicating weak effects on voltage-gated currents (Fig. 2, *B2* and *B3*). The harmonic content of sP waveforms was studied by power spectrum density (PSD) analysis in the 0.02- to 5-Hz bandwidth. In the presence of Ba^2+^, PSD decreased at all frequencies (Fig. 2*B4*).

We next studied the relation between sP amplitudes and spiking frequency. We first analyzed short spike trains and the corresponding interspike intervals (ISIs). Trains of three (ISI = 15.3 ± 2.1 ms; *n* = 21), four (ISI = 17.2 ± 2.4 ms; *n* = 21), or five spikes (ISI = 18.3 ± 3.1 ms; *n* = 12) were followed by sPs having decreasing peak amplitudes (from −22 to −12 μV, respectively), suggesting that the sP amplitude is correlated with the burst frequency. Moreover, we divided the sP by ISI (sP_ISI_) after a 0.4-s rightward shift (Fig. 2*A2*). The sP_ISI_ absolute values (Fig. 2*A3*) reflect the astrocyte K^+^ current amplitudes relative to the burst ISI and suggest that the K^+^ current amplitude depends on a cumulative effect of [K^+^]_o_, whose diffusion is slow compared with the spike timing within bursts.

To study whether our signals changed with time in vitro, we plotted sPs and the corresponding timestamps at different DIV (Fig. 2*C*). The sP amplitude increased with time, remaining synchronized with the spike trains, and was highly sensitive to network disinhibition by GZ. All sPs were negative, except at 1 DIV. This change of polarity is discussed later. Figure 2*D* shows the PSD data obtained from five electrodes (representative of 48). The PSD peaks (located in the 0.1- to 1-Hz region) progressively increased from 1 to 16 DIV (Fig. 2*D*) because of higher neuronal activity and astrocyte proliferation. GZ increased cell firing and sP amplitudes by one order of magnitude.

#### High amplitude of sP signals in organotypic slices.

The narrow extracellular interstices permitted higher spike-dependent accumulation of [K^+^]_o_ compared with primary cultures, leading to much ampler sP signals. The corresponding PSD analysis (Fig. 2*D*) is shown for one experiment (representative of 4) at 16 DIV, in the absence (open circles) or presence (filled circles) of GZ. The peak PSDs computed in dissociated networks (18 μV^2^/Hz) was about 50 times as small as in organotypic networks (900 μV^2^/Hz). We conclude that the spike-related time-dependent [K^+^]_o_ changes are reflected by the sP signals observed in primary cultures as well as organotypic slices.

#### Blocking astrocyte proliferation strongly impaired sP signals in primary cultures.

**Fig. 3.**
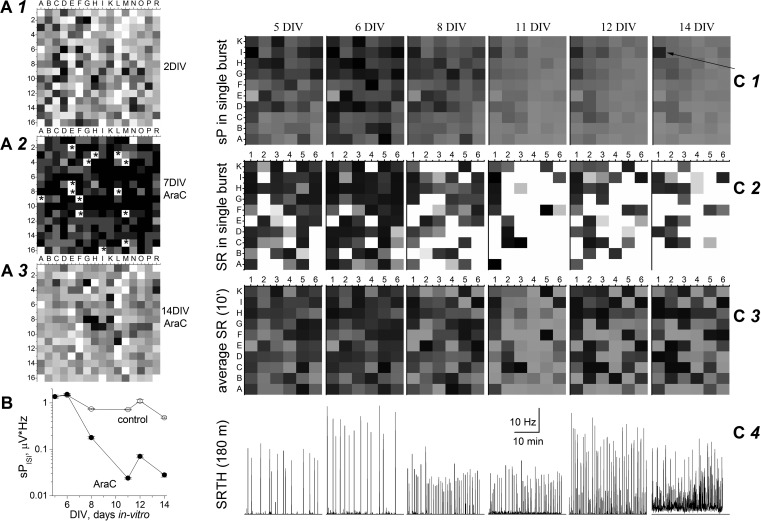
Blocking cell proliferation with AraC inhibits sP signals in primary cultures. In each image, letters (*x*-axes) and numbers (*y*-axes) identify each electrode. *A1–A3*: sP activity in a MEA256 dish (representative of 4) at 2 DIV (*A1*; no AraC) and (in the presence of AraC) at 7 (*A2*) and 14 DIV (*A3*). Gray tones result from linear conversion of sP values ranging from −80 (black) to 0 μV (white) during 100 ms at burst peak. Stars indicate defective electrodes (the 4 corners contained no electrodes). *B*: sP_ISI_ at different time points, calculated from 2 dishes (of 6) prepared from the same mouse litter and treated or not with AraC. Data points in control (open symbols) and in the presence of AraC (filled symbols) are averages of at least 20 electrodes and the following number of bursts (AraC in parentheses): 22 (12), 36 (13), 37 (39), 28 (42), 141 (63), and 89 (56) at the indicated DIV. *C1*: images show sP amplitudes at the peak of single bursts throughout a 3-mm × 5-mm dish. Black and white correspond to −40 and +2 μV, respectively. The arrow at 14 DIV indicates the position of a survived astrocyte (electrode I1) still carrying out [K^+^]_o_ buffering. ANOVA of sP signals shows the differences between images were statistically significant (at *P* < 0.0001) except for the following pairs: 12 DIV–14 DIV, 11 DIV–14 DIV, 11 DIV–12 DIV, and 5 DIV–6 DIV. *C2*: SRs at the peak of single bursts. Black and white correspond, respectively, to 100 and 2.2 Hz, according to an approximate inverse relation to SR. *C3*: average SR activity during 10 min of continuous recording. Grayscale is as defined in *C2*. ANOVA of SR data shows that only 6 of the 15 image couples were statistically different (*P* < 0.05): 14 DIV–6 DIV, 14 DIV–11 DIV, 11 DIV–5 DIV, 11 DIV–6 DIV, 11 DIV–8 DIV, and 11 DIV–12 DIV. *C4*: SR time histograms (SRTH) of the I1 electrode, during 1,800 s of continuous recording, at the indicated DIV. Each vertical line corresponds to a burst reverberating throughout the network. Data were computed at 1-s bins (running average with 3-point smoothing) using the same scales. The ground electrode was E1.

To assess the glial contribution to sPs, we blocked astrocyte proliferation at 3 DIV with AraC (Ahlemeyer and Baumgart-Vogt 2005). The time course of AraC action is shown in Fig. 3. To better visualize the spatial distribution of the effect, the sP amplitudes recorded by different MEA electrodes were converted to a linear grayscale, where black and white corresponded to maximal and zero sP amplitude, respectively. Data were acquired by using 252-electrode MEAs (electrode spacing 200 μm) at the peak of a single burst (0.1 s). Between 2 DIV (Fig. 3*A1*) and 7 DIV (Fig. 3*A2*; i.e., before the onset of AraC action), the dark electrodes progressively prevailed, indicating widespread sP responses. Subsequently, sP amplitudes decreased until 14 DIV (Fig. 3*A3*). Such process was quantified by plotting the average sP_ISI_ (absolute values) at different times in vitro for cultures treated (filled circles) or not (open circles) with AraC (Fig. 3*B*). The control sP_ISI_ was almost constant at ∼1, whereas AraC led to sP_ISI_ progressively decreasing to values 50 times as small. The effect of AraC was not accompanied by a comparable decrease of neuronal activity. Figure 3, *C1–C4*, compares the results obtained from a 3-mm × 5-mm dish (electrode spacing 500 μm), which allowed to sample the largest possible network. AraC was applied at 6 DIV. Images show, at the indicated DIV, the sP spatiotemporal pattern (Fig. 3*C1*), the spike rates in single bursts (SR; Fig. 3*C2*), the average SR (within a 10-min window; Fig. 3*C3*), and the SR time histogram (SRTH; from 180-min continuous recordings; Fig. 3*C4*). Although weakened, action potential firing continued throughout the AraC-treated dishes, whereas sP amplitudes progressively decreased. The corresponding SRs were plotted using a similar grayscale (Fig. 3*C2*). Most neurons were engaged by typical bursts at 5 and 6 DIV, whereas neuronal synchrony decreased between 8 and 14 DIV. To exclude the possibility that this result was peculiar to the chosen burst, we plotted the SR data averaged over a 10-min interval (Fig. 3*C3*). Despite local fluctuations, the overall neuronal activity remained almost uniform at the different time points, suggesting that most neurons had remained viable. A statistical comparison of sP and SR data is given in the legend to Fig. 3.

The long-term effect of AraC on neuronal firing was further characterized on electrode I1, which remained active throughout the dish lifetime (336 h). The corresponding SRTHs are reported in Fig. 3*C4*, with spontaneous reverberating bursts appearing as vertical lines. Burst frequency increased in the presence of AraC, with SR strongly decreasing between 8 and 11 DIV. At 12 DIV, and more so at 14 DIV, the neuronal activity turned into a quasi-continuous firing, which was never observed in untreated cultures. We attribute this result to the progressive impairment of the rapid glia-dependent [K^+^]_o_ buffering after intense spiking activity. In conclusion, although the average neuronal activity was not greatly changed by treatment with AraC, the sP signals were strongly reduced by blocking glial proliferation, in agreement with the notion that the slow signals are essentially caused by astrocyte activity.

#### The presence of inward and outward sPs in neuronal cultures and organotypic slices was consistent with the assumptions of the spatial K^+^ buffering model.

**Fig. 4.**
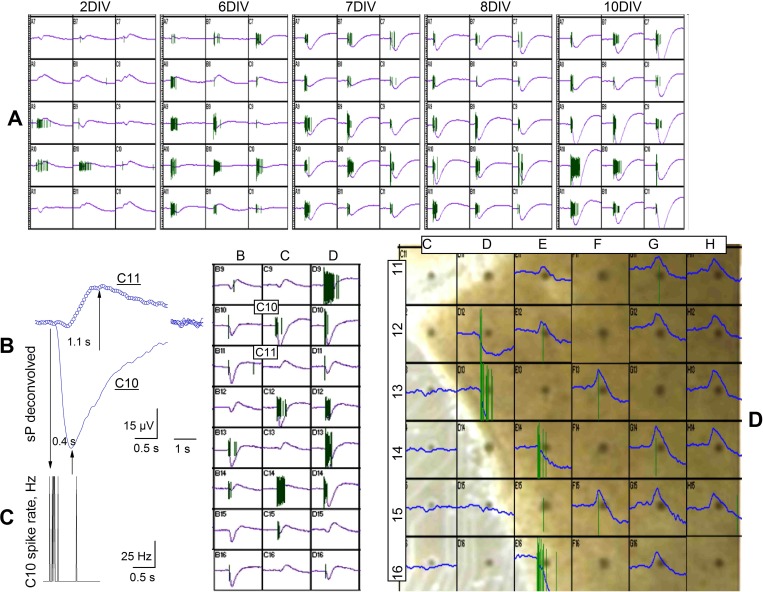
sP signals reflecting inward and outward K^+^ currents in primary cultures and organotypic slices. *A*: sP traces from 15 adjacent electrodes at the indicated age in vitro (representative of 4 MEA256 dishes). The corresponding spikes are shown on top of each trace. *Y*-axes are ±100 μV, and *x*-axes, 5 s. *B*: superimposed sPs showing negative (electrode C10; continuous line) and positive (electrode C11; open circles) deconvolved waveforms, derived from the *right inset*. The latter shows a screenshot of a burst recorded at 2 DIV (24 electrodes are shown from a MEA256 dish; *x*-axes, 5 s; *y*-axes, ±100 μV). Vertical arrows indicate, from *left* to *right*, the first spike, the sP negative peak in electrode C10, and the positive sP peak in electrode C11 (notice the *x*-axis break). *C*: SR computed during the spike train in electrode C10 (bin, 10 ms). Plot is aligned with that in *B*. *D*: screenshot of a representative local burst from an organotypic slice at 2 DIV. In each box, sPs and spikes are superimposed (*y*-axes, ±20 μV; *x*-axes, 2 s). Nondeconvolved waveforms are shown. Electrodes (dark disks in box centers) are identified by letters (columns) and numbers (rows). In electrodes D13 and E16, the negative sPs are off scale. Notice that all the electrodes on the *right* region (F–H columns) display delayed positive sPs. Some electrodes gave no signal because of poor adhesion to the tissue slice.

The spatial K^+^ buffering concept assumes that the firing-dependent [K^+^]_o_ excess is absorbed locally and released at a distance by the astrocyte network (Kofuji and Newman 2004; Orkand et al. 1966). Accordingly, electrodes devoid of spikes should display positive sPs (indicating K^+^ release), with a precise timing relation with the corresponding negative signals (K^+^ absorption). In neuronal cultures, we studied this process in adjacent electrodes (Fig. 4*A*): *1*) at 2 DIV, most electrodes recorded positive sPs, because spike trains were rare; *2*) after 7 DIV, all sPs were negative, because brief spike trains were observed in every electrode; and *3*) meanwhile, the negative sP amplitudes grew by more than one order of magnitude. A detailed characterization was performed in early cultures with a low astrocyte number. At 2 DIV, ∼98% of the electrodes displayed an average SR of 0.024 ± 0.0022 Hz (*n* = 242 ± 3 active electrodes in each dish; 10 dishes were analyzed). Because synapses were immature at this stage, random and coordinated spiking was respectively observed in 34.8 ± 0.3% and 65 ± 2% of the electrodes (10 different sP comparisons were carried out in 10 different bursts). Typical sP responses to a single burst in two adjacent (200 μm) electrodes are shown in Fig. 4*B*. Spikes were present in electrode C10 (line) but not C11 (circles). The SR of C10 is shown in Fig. 4*C*. The negative sP peak (C10) was delayed from the spiking start by 0.4 s, whereas the positive sP peak (C11) was delayed by ∼1.1 s, suggesting distinct but temporally linked current sources. A similar pattern was observed in other pairs of adjacent electrodes (*right inset*; e.g., in B9, B12, B14, C9, C13, C16, and D15).

A similar pattern was observed in organotypic slices. The response of 36 adjacent electrodes to a local burst at 2 DIV is shown in Fig. 4*D*. The electrodes covered by the slice on the *bottom left* region display negative sPs and spike trains (D12, D13, E14, and E16), whereas the active electrodes located on the *right* area show positive sPs with no spike trains. These data are consistent with the hypothesis that sP signals reflect K^+^ handling and that effective spatial buffering may be present in neuronal cultures and brain slices since early stages.

#### “Miniature” slow potentials revealed the [K^+^]_o_ changes induced by single spikes in early primary cultures.

**Fig. 5.**
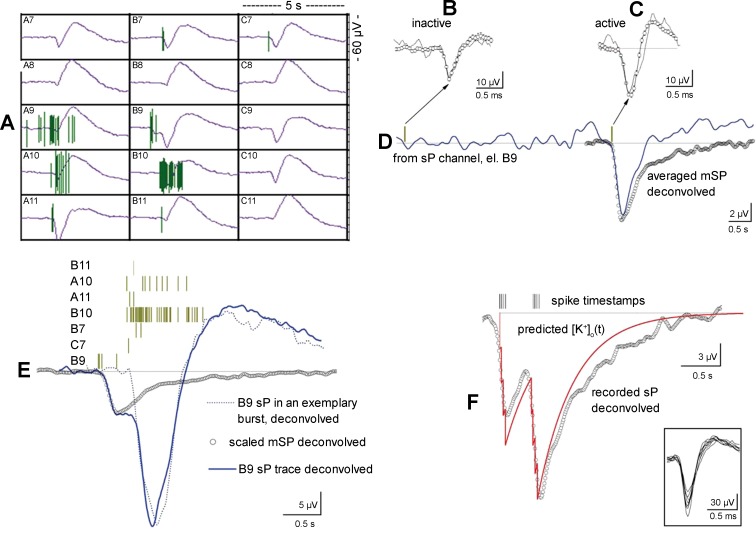
Miniature sPs (mSPs) identify the astrocyte response to single action potentials. *A*: representative waveforms from the electrodes (A7 to C11) surrounding B9 (analyzed in *B–E*). Vertical lines are spikes; blue lines are sP traces. Statistical analysis showed that spiking activity in A9 and B9 was uncorrelated, indicating that A9 and B9 neurons did not directly communicate. Data were acquired with MEA256 at 2 DIV. *B* and *C*: averages of small (*B*; *n* = 15) and large spikes (*C*; *n* = 12). Dotted lines are the spikes occurring at the timestamps indicated in *D*. el, Electrode. *D*: representative sP signal at B9 and 2 timestamps. Open circles are averages of 12 msPs, recorded during 1 h (deconvolved). No msP was ever observed after a small spike (*n* = 15). *E*: the nondeconvolved atypical B9 event shown in *A* was reconstructed by superimposing the following deconvolved waveforms: *1*) the sP signal (blue), consisting of an early msP, caused by a single large spike, and a subsequent sP caused by the global burst; *2*) the scaled average msP (open circles; same as in *D*); and *3*) a scaled typical sP (dotted line). The spike timestamps from 6 other adjacent electrodes are also indicated: only 1 of the 4 B9 spikes was of the large type. *F*: comparison between the theoretical [K^+^]_o_(*t*) curve (red line) resulting from the ISI values (20, 22, 28, 320, 19, 26, and 30 ms; spike timestamps are also indicated) and the left-shifted (0.2 s) experimental sP trace (open circles; after scaling and deconvolution), relative to the double spike train. *Inset*: superimposed spike waveforms during the double train.

We next sought to record the astrocyte depolarization related to a single spike by the same electrode in early primary cultures (to have low network activity). Observation of a miniature slow potential (msP) is expected to be a rare event, because the axon hillock and the astrocyte must be located not farther than 40 μm from the border of the electrode (Pettersen and Einevoll 2008). Moreover, the neuron must fire more frequently during the intervals between global bursts, when [K^+^]_o_ is at the steady state. Nonetheless, msPs were observed in 2 different electrodes among 6 dishes at 2 DIV. One of these (B9) is shown in Fig. 5*A*, along with the adjacent electrodes. The B9 spikes constituted two well-separated amplitude classes, whose averages (circles) are shown, respectively, in Fig. 5, *B* and *C*. Two individual spike waveforms are superimposed for comparison (dotted lines). Only the largest spikes (*n* = 12) were followed by an msP, suggesting that in these cases the neuron-astrocyte pair was sufficiently close to the recording electrode. A representative sP signal (blue trace; nondeconvolved) displaying an msP occurring after a large, but not a small, spike (indicated by vertical red timestamps) is shown in Fig. 5*D*. The result of averaging all the deconvolved msPs is superimposed (circles). The average delay between the large spike and the corresponding msP peak was 0.2 ± 0.01 s (*n* = 12). An expanded illustration is shown in Fig. 5*E* (deconvolved traces), showing both the msP (circles) and a typical global sP (dotted line). The sum of the two traces broadly corresponds to the acquired sP (continuous blue line).

Overall, at early in vitro stages, positive sPs prevail because many neurons are silent during global bursts (see also Fig. 4*A*). Moreover, the acquired sPs can reflect both early fast inward and late slow outward K^+^ currents (e.g., electrodes A11, B7, C7, and B9), suggesting that an individual electrode can sample a relatively wide astrocyte area. In contrast, the msPs could only be recorded as inward events, probably because of their small amplitude.

#### The kinetics of [K^+^]_o_ change.

Frankenhaeuser and Hodgkin (1956) represented the glial response to the spike-dependent [K^+^]_o_ increase as a superposition of elementary decaying currents. In the CNS, we assumed such exponential decay has a time constant τ = d*x*/*P*_K_, where *P*_K_ is the K^+^ permeability of astrocytes and d*x* is the distance between the neuronal and astrocyte membranes. Fitting a monoexponential curve to the decaying average msP of Fig. 5*D* gave τ = 0.61 ± 0.008 s (*R*^2^ = 0.96). This procedure was also applied to the sP deriving from a double burst (Fig. 5*F*). The red line describes the model-derived [K^+^]_o_(*t*) during neuronal activity, which is proportional to the underlying K^+^ current. The scaled experimental sP is also shown (open circles). The qualitative match between the sP trace and the theoretical [K^+^]_o_(*t*) curve indicates that the Frankenhaeuser-Hodgkin model can be directly applied to neocortical networks. Our results show that the astrocyte response to the spike-dependent [K^+^]_o_ change rises very quickly (on the order of 100 ms) and that the entire K^+^ buffering process occurs within a subsecond timescale.

#### Comparing K^+^ and GluT currents in primary cultures.

**Fig. 6.**
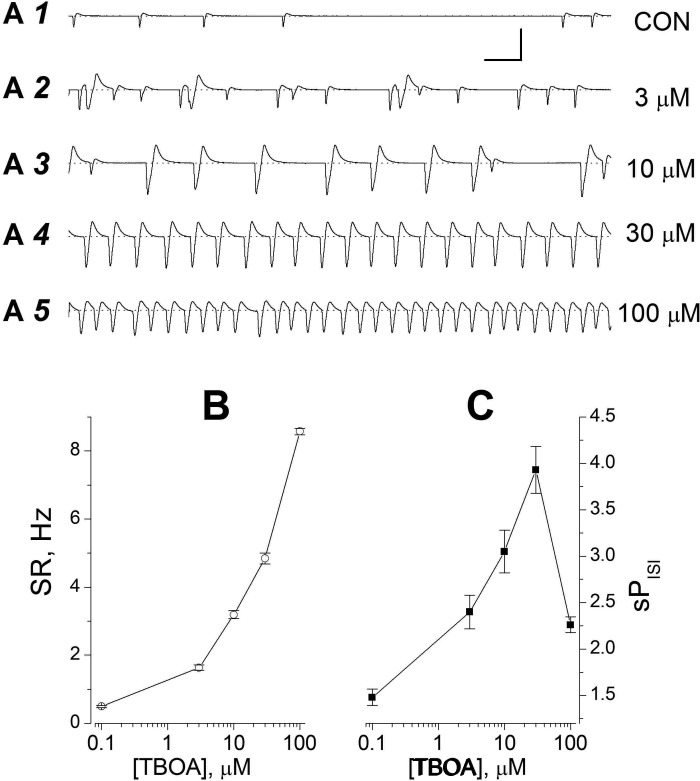
Blocking GluT with TBOA causes hyperexcitability and sP upsurge in primary cultures. *A1–A5*: sP traces (150 s) in the absence (CON) or presence of the indicated TBOA concentration, applied for 10 min, at 13 DIV. Data are representative of 48 electrodes. Scale bars, 10 s and 50 μV. *B* and *C*: SR (*B*; open circles) and sP_ISI_ (*C*; filled squares) vs. TBOA concentration (log scale; the control was arbitrarily set at a virtual TBOA concentration of 0.1 μM).

**Fig. 7.**
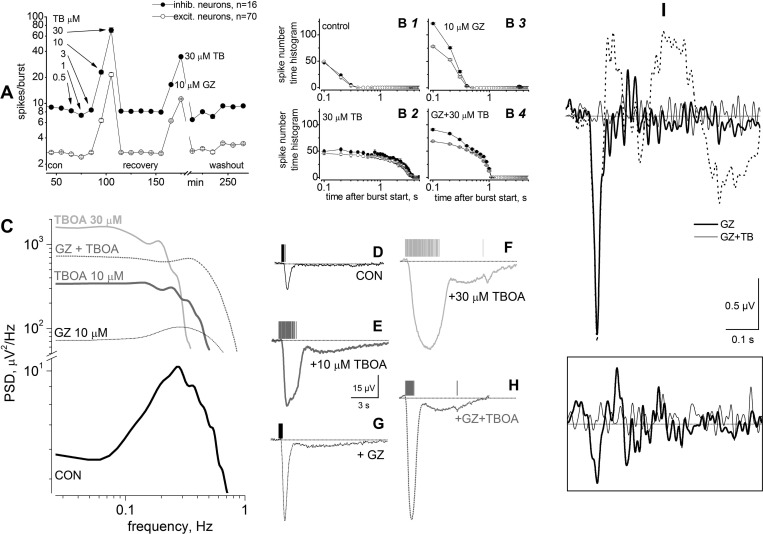
GluT currents are blocked by TBOA and enhanced by GZ in primary cultures. *A*: number of spikes per burst calculated from 1 MEA dish (of 4) for inhibitory (filled symbols) and excitatory neurons (open symbols). Data points are averages calculated for 10-min intervals. Drugs were applied for 10 min. Notice the log scale on *y*-axis and the break on x-axis (time) before washout. *B1–B4*: spike number time histograms (SNTH) evaluated in control (*B1*) and in the presence of 30 μM TBOA (*B2*), 10 μM GZ (*B3*), and GZ + TBOA (*B4*). The drugs produced different effects on the time course of elicited spikes and burst duration. *C*: PSD analysis of the sPs relative to the data illustrated in *A*, calculated for 500-s continuous recording in control (CON) or in the presence of the indicated drugs. *D–H*: representative sP waveforms and the corresponding spike timestamps. From CON conditions (*D*), TBOA was added at 10 μM (*E*) and then 30 μM (*F*). After 2 h of recovery, 10 μM GZ was added (*G*), and finally, 30 μM TBOA was added in the presence of GZ (*H*). Notice the small response to a few spontaneous spikes (*F* and *H*) toward the end of the burst. *I*: averaged deconvolved LFP waveforms from the same electrode in the presence of 30 μM GZ (thick line; average of 63 traces) and 30 μM GZ + 30 μM TBOA (thin line; average of 85 traces). A nondeconvolved LFP trace (dotted line; in GZ) is shown for comparison. Waveforms were statistically different in both conditions (*t*-test, *P* < 10^−5^). *Inset*: same analysis in control (thick line) and TBOA (thin line).

GluT currents precede the long-lasting Ba^2+^-sensitive K^+^ current and are selectively blocked by TBOA (Bergles and Jahr 1997; Diamond et al. 1998; Lüscher et al. 1998; Sibille et al. 2014). Because GluT (particularly GLT-1) expression strongly increases in the first 10 days of primary hippocampal cultures (Perego et al. 2000), we applied TBOA from 11 to 13 DIV (*n* = 3; Fig. 6) and 19 to 21 DIV (*n* = 4; Fig. 7). TBOA was applied at 3 to 100 μM, to span the reported range of effective concentrations (Campbell and Hablitz 2008; Shimamoto et al. 1998). Even the lowest concentration considerably increased the amplitude and duration of the small and short burst-related sP waveforms, as is clear from comparison of the waveforms in Fig. 6*A1* (controls) with those in Fig. 6*A2* (3 μM TBOA). Higher concentrations also enhanced the frequency (Fig. 6, *A3–A5*), and both SR and sP_ISI_ increased with TBOA concentration. The average SR and sP_ISI_ (absolute values) are plotted, respectively, in Fig. 6, *B* and *C*. The effect was detectable at 3 μM and saturated at 100 μM. At 100 μM, sP_ISI_ decreased because of the considerable increase in bursting rate (Fig. 6*A5*) and the impossibility of recording the full DC amplitude of K^+^ currents (see materials and methods).

To simultaneously study GluT and K^+^ currents at 19 DIV, we first carried out a TBOA concentration-response test and next applied a saturating TBOA concentration after disinhibiting the network with GZ (10 μM). A representative experiment is shown in Fig. 7*A*, plotting the average number of elicited spikes in each burst, computed in 10-min intervals. The results are given for both excitatory (∼3 spikes) and inhibitory (∼10 spikes) neuronal clusters. After 90 min in control condition, we applied the indicated TBOA concentrations, which proportionally increased the number of spikes per burst. After recovery, GZ was applied. The ensuing network unbalancing led to ∼6 (excitatory cluster) and ∼20 (inhibitory cluster) spikes/burst. In the presence of GZ, TBOA further increased spiking activity to ∼10 and ∼40 spikes/burst, respectively. The effect was reversible on washout. The corresponding SNTHs are displayed in Fig. 7, *B1–B4*. The histograms relative to the short burst duration were similar in the two neuronal clusters in control conditions (Fig. 7*B1*), because in a balanced network the total number of spikes generated by the few fast-spiking inhibitory cells is approximately similar to the number produced by the many slow-firing excitatory cells. TBOA (Fig. 7*B2*) strongly increased the burst duration (from 0.2 to 2 s), whereas GZ (Fig. 7*B3*) doubled the spike number without increase in burst duration. In the presence of both drugs (Fig. 7*B4*), a mixture of effects was observed, because of the combined action on GluT and GABAergic synapses. In the presence of GZ, higher glutamate concentrations are found near the excitatory synapses, which cause larger GluT astrocytic currents that are totally blocked by TBOA. The corresponding effect on K^+^ currents is shown by PSD plots (Fig. 7*C*) and the deconvolved sP traces (Fig. 7, *D–F*), which were considerably increased by TBOA, as expected.

GluT currents were identified by averaging LFP waveforms (captured from ∼0.1 s before each burst). Comparing the trace recorded in the presence of 10 μM GZ (Fig. 7*I*, thick line) with those obtained when 30 μM TBOA was added (Fig. 7*I*, thin line) shows that a large transient inward current was synchronized with each global burst. The nondeconvolved LFP signal is indicated by the dotted line. In the absence of GZ (*bottom inset*), the effect of TBOA was smaller, because the network activity was much weaker (Fig. 7*A*).

#### Simultaneous recording of spikes, GluT currents, and K^+^ currents in organotypic networks.

**Fig. 8.**
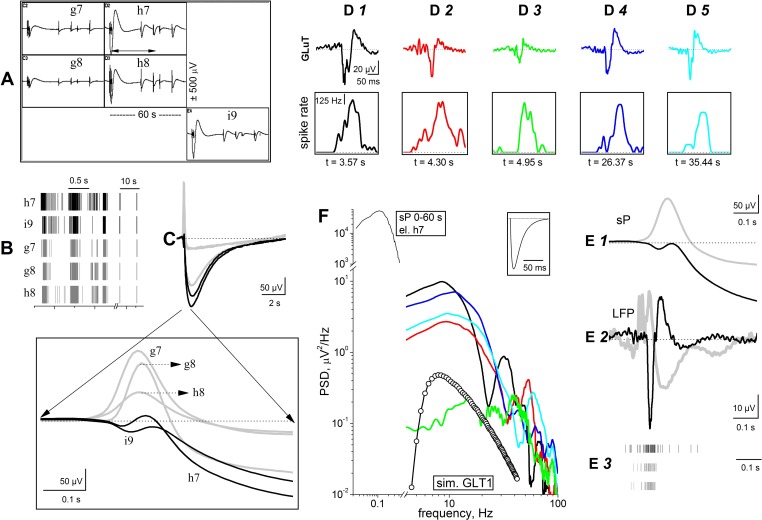
Simultaneous recording of spikes, GluT current, and K^+^ current in organotypic slices. *A*: sP waveforms and spikes from the indicated adjacent electrodes sampling a PFC region (representative of 10 MEA256 dishes). For all electrodes, *x*- and *y*-scales are, respectively, ±500 μV and 60 s. More than 2 spiking neurons were identified in each electrode. *B*: raster plot of the timestamps recorded from each electrode, from 3 to 38 s (see horizontal arrow in *A*). Notice the *x*-axis break and the change in scale at 5.5 s. *C*: deconvolved sPs relative to the second burst (at 26.4 s) for the 5 electrodes. The first 0.6 s of each trace is further expanded in the *inset*. The gray traces (electrodes g7, g8, and h8) displayed an early positive upstroke; the black ones (electrodes h7 and i9) had a negative-going trend. *D1–D5*: deconvolved LFP waveforms from electrode h7, relative to the bursts starting, respectively, at 3.572, 4.301, 4.955, 26.374, and 35.449 s. The peak amplitudes of the first 3 traces were ∼−52 to ∼−39 and ∼−19 μV; the corresponding time integrals were, respectively, −660, −326, and −320 ms. *Insets*: corresponding SRs calculated in the same time interval (bin, 10 ms). In this electrode, at least 3 neurons were identified. *E*: in all panels, aligned traces were obtained from the burst at 26.4 s (*inset* in *C*). In *E1* (sPs) and *E2* (LFPs), gray traces are averages of g7, g8, and h8, whereas black traces are averages of h7 and i9. *E3*: spike timestamps for h7 (*top*), g7 (*middle*), and g8 (*bottom*). *F*: PSD analysis of the data relative to waveforms in *D* (lines; same colors as in *D*) and the waveform shown in the *inset* (lines + open symbol). Notice the *x*- and *y*-axis breaks. In the *top left* corner, the PSD of the 60 s of data from electrode h7 is shown. *Inset*: simulated theoretical curve representing a typical GluT current. Simulation was performed with exponentials with trailing and decaying time constants of 3 and 26 ms; peak amplitude was 50 μV.

The sP and LFP amplitudes measured in organotypic networks exceeded those observed in primary cultures by several orders of magnitude. Hence, a single burst could elicit a measurable GluT current, avoiding the necessity of averaging hundreds of events and using pharmacological tools. As expected, only the electrodes displaying large negative sPs also showed simultaneous GluT currents. An example is shown in Fig. 8*A*, showing sP traces from adjacent electrodes (g7 to i9), during 1 min in which five bursts with different IBIs were observed. The timestamp structure of the first three bursts is shown in Fig. 8*B* (notice the *x*-axis break at 5.5 s). The first consisted of three sub-bursts with short IBIs and was f ollowed by two briefer bursts at ∼26 and ∼35 s. Because of the short IBIs, the sP amplitudes related to the first burst were considerably larger than the subsequent ones, in agreement with the results obtained in primary cultures (Fig. 2). The firing frequency was considerably higher in h7 and i9 than in g7, g8, and h8 electrodes (as indicated by the timestamps' gray level). The five deconvolved sP waveforms corresponding to the second burst (at ∼26 s) are expanded and superimposed in Fig. 8*C*. The trace decay with an ∼2-s time constant is consistent with patch-clamp results (De Saint Jan and Westbrook 2005). On the contrary, the early response (expanded in the *inset*) shows that g7, g8, and h8 had an early positive peak, whereas g7 and i9 displayed the usual negative waveform, with an ∼200-ms delay. Therefore, these sPs derived either from astrocytes located in different regions or from the summation of K^+^ currents from different areas of a large astrocyte covering more than one electrode.

Because electrode h7 displayed the quickest negative sP, it was likely the closest to the nearby firing neurons. In this electrode, for the bursts shown in Fig. 8*B*, we studied the GluT-related LFPs for the five bursts (Fig. 8, *D1–D5*; after deconvolution). The corresponding SR plots are given in the *insets*. The GluT current peak generally preceded the SR peak by ∼50 ms. The first three GluT traces showed a progressive amplitude decline, which recovered in later bursts (at 26.4 and 35.5 s). Such a behavior was often observed during consecutive bursts (see discussion). The brief negative GluT currents were not observed in the LFP channels from electrodes g7, g8, and h8, suggesting that these electrodes did not sense a [K^+^]_o_ increase but detected a glutamate increase sufficient to activate GluT. The average sP and GluT currents from the electrodes giving early negative (black) or positive (gray) responses are given, respectively, in Fig. 8, *E1* and *E2*. The corresponding spike burst timestamps are aligned in Fig. 8*E3*. As expected, GluT currents slightly preceded the spike trains. Moreover, in the time segment normally occupied by GluT currents, the average LFP signal of the electrodes with positive sPs showed no fast negative current (see Fig. 8*E2*).

Finally, GluT currents were expected to give peak PSD values intermediate between those of spikes (below 0.02 μV^2^/Hz) and sP signals (around 50,000 μV^2^/Hz). Figure 8*F* plots the superimposed PSDs corresponding to the GluT data of Fig. 8*D* (same trace colors), showing that PSD did not exceed 10 μV^2^/Hz. For comparison, we also superimposed (line + open circles) the PSD corresponding to a transient inward current (see *inset*) that best approximates the GluT current waveform recorded in hippocampal slices (Diamond 2005) and the PSD of an sP (for electrode h7) during the 60-s time interval. Similar results were obtained in other regions, as well as other dishes, between 4 and 15 DIV. In conclusion, in brain slices, action potentials, GluT currents, and Kir currents can be simultaneously captured by individual MEA electrodes.

## DISCUSSION

In the CNS, a long-term steady [K^+^]_o_ is maintained by the relatively slow action of the Na^+^ pump (e.g., D'Ambrosio et al. 2002). However, high neuronal activity can cause a local [K^+^]_o_ increase that cannot be rapidly reabsorbed by the Na^+^ pump. On the basis of studies in leech and salamanders, Orkand et al. (1966) proposed that such excess K^+^ could be locally absorbed by glial cells, because the K^+^ equilibrium potential across the glial membrane transiently depolarizes compared with resting potential. The syncytial nature of glial tissue would then allow K^+^ to be released from cells located far from the active focus, where [K^+^]_o_ has remained at the resting concentration. Such a “spatial buffering” mechanism has been thoroughly investigated in the retina and related structures (Kofuji and Newman 2004). In retinal Müller cells, the model has been extended by the observation that asymmetric distribution of K^+^ channels along the cell membrane (Newman 1984) allows “siphoning” of excess K^+^ to the vitreous substance (Brew et al. 1986; Newman et al. 1984). These mechanisms are often assumed to be operant in other CNS regions but are still not demonstrated (Ransom 1996). Nonetheless, recent work in hippocampal astrocytes has demonstrated syncytial isopotentiality ensuing from strong electrical coupling between astrocytes (Ma et al. 2016). To capture the different electrophysiological aspects of this mechanism in neocortical tissue, we extended the MEA method to study the spatiotemporal properties of the neuron-glia cross talk in large networks (∼30,000 cells for ∼10 mm^2^) and sample for weeks the cellular responses at timescales ranging from submilliseconds to minutes.

### 

#### Measuring astrocytic K^+^ currents with MEAs.

The observation of spontaneous and burst-linked negative msPs shows that astrocytes can respond to the small [K^+^]_o_ change produced by a single action potential. Moreover, the spike-related Ba^2+^-sensitive negative sPs were tightly linked to the positive sP waveforms recorded from farther electrodes. At early in vitro stages, sPs were mainly positive (reflecting outward K^+^ currents), because spike trains were rare. Between 2 and 10 DIV, the frequency of positive sP events decreased, whereas the amplitude of negative sPs dramatically increased, along with SR and burst frequency (see also Sun et al. 2010). The presence of sP waveforms of opposite polarity matching the local presence or absence of spikes is consistent with the assumptions of the spatial K^+^ buffering model, which is based on the electrical syncytial properties of the glial network (Kofuji and Newman 2004; Ma et al. 2016). Moreover, the observation that fast negative and late positive sPs were detected with spatiotemporal precision of 200 μm/0.7 s (Fig. 4, *B–D*) and 40 μm/0.2 s (Fig. 5*E*, same electrode) suggests that our method provides a way to investigate the mechanism of K^+^ siphoning to nearby regions (Kofuji and Newman 2004; Wallraff et al. 2006). Major evidence that sPs are essentially astrocytic events is the effect of AraC. When glial proliferation was impaired, the tight correlation between SR and sP signals was strongly reduced, and sPs almost disappeared by 16 DIV. Nonetheless, neuronal firing persisted throughout the network, although weakened, indicating that AraC had not caused widespread neuronal damage.

#### Glutamate reabsorption and GluT currents.

The synaptically released glutamate is reabsorbed very quickly, as is necessary for fast control of microcircuit function (Auger and Attwell 2000; Diamond 2005; Diamond and Jahr 1997). Moreover, the steady nonvesicular glutamate release also must be tightly controlled, to avoid neurotoxic effects (Danbolt 2001; Rosenberg et al. 1992; Zhou and Danbolt 2013). In fact, blocking glutamate reabsorption causes significant extracellular accumulation of the neurotransmitter (Jabaudon et al. 1999). The action of TBOA was beautifully described in olfactory bulb glomeruli, where fast GluT and slow Kir astrocyte currents were simultaneously recorded on glutamatergic stimulation (De Saint Jan and Westbrook 2005). The abnormal extracellular levels of glutamate caused by GluT inhibition can also induce hyperexcitability by activating metabotropic glutamate receptors in olfactory bulb (De Saint Jan and Westbrook 2005), spinal cord dorsal horn (Galik et al. 2008), hippocampus (Molinari et al. 2012), and midbrain periaqueductal gray (Wilson-Poe et al. 2013).

In our networks, high TBOA concentrations abolished the normal reverberating activity and strongly increased the burst length in both excitatory and inhibitory neuron clusters. This is consistent with previous observations in physiological conditions (De Saint Jan and Westbrook 2005), as well as with studies showing that seizures are induced by TBOA in developing rat neocortex (Demarque et al. 2004), in mice lacking GLT-1 (Aida et al. 2015), and in seizure-susceptible mice (Inyushin et al. 2010). The GluT waveforms were always synchronized with both spikes and sPs, suggesting that our experimental model allows direct study of how the underlying mechanisms interplay. The decrease observed in GluT current amplitude during consecutive bursts (Fig. 8, *D1–D3*) could depend on partial local depletion of releasable glutamate (Jones et al. 1997), possibly accompanied by alteration of uptake kinetics because of the progressive involvement of different transporter pools (Diamond 2005). In principle, the LFP channel filtering we adopted should permit record both GluT and synaptic currents. However, only the large GluT currents turned out to be detectable, probably because the two above signals are largely superimposed (Murphy-Royal et al. 2015). Finally, although we attribute most of the presented effects to astrocyte currents, the possible involvement of microglia (Gullo et al. 2014a) and oligodendrocytes (Maldonado et al. 2013) merits further investigation.

#### Conclusions.

MEA recording couples the high sensitivity of electrical detection with the possibility of sampling a wide area. Moreover, cell culture activity can be followed for weeks, which potentially permits insight into developmental and pathogenetic mechanisms (Chiappalone et al. 2006; Gullo et al. 2014b; Hammond et al. 2013; Hogberg et al. 2011; Wagenaar et al. 2006). Application of our methods to other excitable tissues, such as the muscular and endocrine, should be relatively straightforward, because in these tissues the whole cell current amplitudes are of the same order as those observed in the nervous system. Less obvious is how to apply these methods to nonexcitable tissues, including the neoplastic, which may display much smaller cell current amplitudes. An order of magnitude estimation is as follows. The sP and LFP signals depended on K^+^ and GluT currents, respectively, with amplitudes usually ranging from hundreds of picoamperes to several nanoamperes (De Saint Jan and Westbrook 2005; Diamond 2005). Whole cell K^+^ currents of similar or higher amplitude are easily observed, for instance, in human glioma explants (Becchetti et al. 2002; Masi et al. 2005), whereas GLT-1 is strongly reduced (Ye et al. 1999). Hence, the neuron/astrocyte/glioma interplay at the boundary of normal and neoplastic tissue should be amenable to analysis, at least as far as K^+^ buffering is concerned. Regardless, our approach should facilitate deeper studies about the dynamics of local and global ionic mechanisms in the homeostasis of cerebrospinal medium and about the regulation of synaptic function, for which a fully coherent picture is missing (Nedergaard and Verkhratsky 2012).

## GRANTS

This work was funded by Italian Telethon Foundation Grant GGP12147 (to A. Becchetti), Cariplo Foundation Grant 2008.2907 (to E. Wanke), and a FAR grant from the University of Milano-Bicocca (to A. Becchetti).

## DISCLOSURES

No conflicts of interest, financial or otherwise, are declared by the authors.

## AUTHOR CONTRIBUTIONS

E.W. conceived and designed research; E.W., F.G., and E.D. performed experiments; E.W. and G.V. analyzed data; E.W., G.V., and A.B. interpreted results of experiments; E.W. prepared figures; E.W. and A.B. drafted manuscript; E.W., F.G., E.D., G.V., and A.B. edited and revised manuscript; E.W., F.G., E.D., G.V., and A.B. approved final version of manuscript.

## ENDNOTE

At the request of the authors, readers are herein alerted to the fact that additional materials related to this manuscript may be found at the institutional website of one of the authors, which at the time of publication they indicate is: http://boa.unimib.it/handle/10281/25492. These materials are not a part of this manuscript, and have not undergone peer review by the American Physiological Society (APS). APS and the journal editors take no responsibility for these materials, for the website address, or for any links to or from it.
